# Discourse markers in TV interviews: A corpus-based comparative study of Chinese and the western media

**DOI:** 10.3389/fpsyg.2022.1063158

**Published:** 2022-12-01

**Authors:** Yanli Fu, Victor Ho

**Affiliations:** ^1^Faculty of Humanities, The Hong Kong Polytechnic University, Hong Kong, Hong Kong SAR, China; ^2^Department of English and Communication, The Hong Kong Polytechnic University, Hong Kong, Hong Kong SAR, China

**Keywords:** discourse marker (DM), DM frequency, DM pattern, DM position, TV interviews, corpus-based comparative study

## Abstract

This article, which is part of an on-going large-scale study, quantitatively explores and compares the frequency, patterns, and positions of the three most frequently used discourse markers (DMs): *so, and, but* in TV interviews. The data comprise three corpora consisting of three media programs from China, the US, and the UK. Results show that there is a statistically significant difference in the frequency of the DM *so* and the DM *and*, with each DM having the highest frequency in a specific corpus. Four co-occurring strings (*“and so,” “and but,” “so but,” “but so”*) are identified in the three corpora with the DM co-occurrence *“and so”* having the highest frequency in the American program, supporting the claim that this combination is a typical use in American English. The general positional distribution of the three DMs is similar with the highest tendency in the initial position, which can be attributed to the program’s interactivity. The findings will enhance our understanding of the three DMs used in media discourse and should be of practical significance to media hosts and guests in achieving better bilateral communication.

## Introduction

Signals, such as discourse markers (DMs), are frequently utilized by speakers in utterances to direct the hearer through the process of interpretation (Foolen, 2011). Lexical expressions that are predominantly produced from conjunctions, adverbials, and prepositional phrases are referred to as DMs within the subclass of pragmatic markers (Fraser, 1996, 2009). Schiffrin (1987) defines DMs as sequentially dependent elements that bracket units of talk. The three monosyllabic DMs—*so, and, but*—have been chosen as the central focus in this study given their high frequency and keyness, as evidenced by spoken corpora (Rühlemann, 2019) such as British National Corpus 64 (BNC64) and British National Corpus 2014 (BNC2014), in which the three selected DMs rank first on the frequency list. Moreover, according to Fraser’s (2009) taxonomy, the three DMs belong to distinct groups; *so* is an inferential discourse marker (IDM); *and* is an elaborative discourse marker (EDM); *but* is a contrastive discourse marker (CDM).

Against this backdrop, the current study aims to explore media talk, specifically TV interviews. Media talk, as a particular genre, provides insights into the nature of mass communication and serves as a bridge between the media, public opinion, and public knowledge. Recently, media talk has begun to be studied as a phenomenon in its own right (Hutchby, 2006). Studies on media talk have been carried out focusing on the spoken discourse, such as radio talk shows (Hutchby, 2006; Tolson, 2006), television talk shows (Ilie, 2001; Lauerbach and Aijmer, 2007), quiz shows (Culpeper, 2005), and web page talk (Kopf, 2022). Created in the 20th century, TV interview, as a semi-institutionalized socio-cultural practice, has grown more popular and received consistently high ratings over the years (Ilie, 2001). This type of program frequently demonstrates stringent host-initiated queries, typically including face-threatening activities such as direct and unpleasant questions (Furkó and Abuczki, 2014). These features are attributable to the program’s discursive and linguistic qualities.

The characteristics of the program reveal the use of a set of pragmatic language realizations, such as the use of discourse markers (DMs) (Furkó and Abuczki, 2014). DMs can process pragmatic inferences by reducing the hearer’s processing effort (Aijmer and Simon-Vandenbergen, 2004; Furkó and Abuczki, 2014). DMs can function on the politeness level (Östman, 1981), phatic level (Aijmer, 2002), as a face mitigator (Crible, 2018), and for weakening the illocutionary force (Leech, 2014). In addition, the high frequency of DMs appearing in spoken genres makes their use a distinctive feature and a pivotal role in spoken English (Carter and McCarthy, 2006; Farahani and Ghane, 2022). TV interview is a type of oral interaction between the host and the guest that provides a good opportunity to examine DMs in spoken discourse (Oyeleye and Olutayo, 2012). As a result, an increasing number of studies have been dedicated to the investigation of DM in media discourse with DM function being the most explored area, such as the mapping of the DM functional spectrum in media discourse (Furkó, 2015), the examination of DM types and functions in mediatized interviews (Furkó and Abuczki, 2014), talk shows (Kang, 2018, 2019), and interview videos (Tsoy, 2022). Despite the widespread interest in DMs, other aspects such as frequencies, patterns, and positions have received less attention in the literature, particularly in the context of TV interviews. The current study, which is a part of an on-going large-scale comparative project examining DMs in media discourse, attempts to contribute to the existing literature by exploring from a quantitative perspective.

## Previous studies

In the past, there has been a great deal of interest in the theoretical study of DMs, with much of that interest focusing on their definition, meaning, and functions. For instance, the majority of the studies tend to advocate for different interpretations of DMs, such as discourse connectives (Blakemore, 2002), discourse particles (Schourup, 1999), and connect a variety of theoretical models, like Redeker’s (1990) model and Schiffrin’s (1987) five distinct planes. The last few years have witnessed an increase in the number of empirical studies examining the role of DMs in various circumstances, such as mediatized institutional political interviews (Furkó and Abuczki, 2014), scientific papers (Rezanova and Kogut, 2015), Asian presidents’ addresses (Banguis-Bantawig, 2019), therapeutic interviews (Cepeda and Poblete, 2006), academic spoken English (Farahani and Ghane, 2022), and laboratory experiments (Holtgraves and Bonnefon, 2017). Another emerging trend in DM research is a growing interest in comparing the use of DMs in terms of functions and frequency between English native speakers (NSs) and non-native speakers (NNSs) from a variety of L1 backgrounds (Müller, 2005; Aijmer, 2011; Asik and Cephe, 2013; Al-khazraji, 2019; Şahin Kızıl, 2021).

Previous research on the three selected DMs has also examined their functions in structural relations, cohesive relations, and interactional relations, particularly their role in achieving discourse coherence. These studies use either natural data, such as sociolinguistic group interviews in which several people are invited to prompt one another to speak (Schiffrin, 1987), a film description experiment in which discourse markers are elicited from native American English speakers’ descriptions of films that they have seen to others without having watched (Redeker, 1990), or constructed examples created by the scholar for the purpose of examining DMs (Fraser, 1999, 2009). According to Huang (2019), corpus methodology is frequently used in DM research, which is supported by Schiffrin’s (1987) interview data, who also proposes that corpus is useful for analyzing discourse markers. Moreover, with the employment of corpus, Schirm (2012) examines Hungarian DM *hát* in semi-guided informal conversations and job interview dialogues; Furkó and Abuczki (2014) compare six DMs (*I mean, of course, oh, well, I think, you know*) in BBC and CNN political interviews; Huang (2019) and Buysse (2020) compare the use of DM *well* in a spoken learner corpus between NSs and NNSs.

Studies on DM *so* can be classified into two types. One is the investigation of the multifunctionality of the DM *so* in various contexts, and the other is primarily employing the comparative approach. The exploration of the function of the DM *so* has been conducted in learner corpora (Buysse, 2007; Algouzi, 2021), in naturally occurring face-to-face and telephone interactions (Bolden, 2008, 2009; Barske and Golato, 2010), in seminar talks (Rendle-Short, 2003), in video-mediated communication (Collet et al., 2021), and in English TV programs (Li and Xiang, 2020). On the other hand, comparative studies on the DM *so* have focused exclusively on comparing *so* with other DMs, while others have been particularly interested in comparing how the DM *so* is used by NSs and NNSs. Bolden (2006) compares the interactional role of the DM *so* and *oh* in a corpus of everyday face-to-face and telephone conversations. Nneka (2022) analyzes and compares the function and frequency of DM *so* and DM *well* in a small corpus of three presidential chats, showing that the two DMs can be used to effectively manage the discourse flow, with the DM *so* occurring more frequently than the DM *well*. Lam (2007) compares the frequency, functions, and positions of the DM *so* and the DM *well* between the Hong Kong Corpus of Spoken English (HKCSE) and British National Corpus (BNC) and discovers that the function and frequency of *so* vary according to text genre. Algouzi (2021) analyzes the frequency of the functional distribution of the DM *so* in the LINDSEI-AR sub-corpus. Some comparative studies on the DM *so* in learner corpora are also reported in Müller’s (2005), Buysse’s (2012, 2014), and Liu’s (2017) studies. Studies examine the function of the DM *and* tend to rely on loosely extracted examples from multiple sources, such as examples retrieved from the spoken learner corpora (Heng, 2005), showing that the DM *and* can serve as an addition, a comparison, and a delaying device. Research on the DM *but* focuses on how it functions across a range of genres and literary forms, such as oral narratives (Norrick, 2001), a diachronic corpus of Northern English conversations (Hancil, 2018), and on comparisons between the DM *but* and its counterparts in other languages (Alsager et al., 2020; Khammee, 2022; Shirzadi et al., 2022).

TV interviews have been studied from the im/politeness, language, and ideological perspectives, such as how im/politeness models and strategies are used (Culpeper, 2005; Cook, 2014; Fedyna, 2016; Rabab’Ah et al., 2019; Damayanti and Mubarak, 2021; Sitorus et al., 2022) linguistic features (Ilie, 2001), the representation of ideologies and power relations (Bilal et al., 2012; Sharifi et al., 2017), the host’s role in managing discourse (Oyeleye and Olutayo, 2012), structural units (Kamil Ali, 2018), and guests’ non-serious responses (Sheikhan and Haugh, 2022), to name but a few. However, as previously stated, few studies have focused specifically on DMs in the context of TV interviews. Notable exceptions are Cook (2014), Fedyna (2016), and Kamil Ali (2018), who indirectly show the DM role and functions. There are a number of additional studies, which, however, focus on the qualitative analysis of the DM functions, seldom do they explore the frequency, patterns, and position from quantitative and comparative perspectives, exceptions can be found in the investigation of Korean DM position (Kim et al., 2021a,b), the examination of co-occurrences of Persian DM *vae*, equivalent to English DM *and* (Kazemian and Amouzadeh, 2022), the DM combination “*and now*” (Shirtz, 2021), the DM sequence “*and so*” and “*so and*” (Koops and Lohmann, 2022), and the frequency of “*so*” (Algouzi, 2021) and *“just so”* (Kaltenböck and Ten Wolde, 2022).

Discourse marker co-occurrence is pervasive and relatively frequent, but little work has been done on their ability to combine (Fraser, 2015), and it has been somewhat overlooked until recently (Cuenca and Crible, 2019). For example, Fraser (2010, 2013) examines the acceptability of CDM in examples drawn from COCA (Corpus of Contemporary American English) and BNC (British National Corpus), as well as discussing general functions of the DM *but*. In addition, Fraser (2015) extends the scope by investigating the combination of CDM and IDM, showing acceptable cases for such combinations. Although Fraser’s studies on DM cluster are insightful, he did not provide satisfactory explanations for such co-occurrences, and he also failed to explore the combination of EDM with the other two types. In view of this, quite a number of underlying motivations for such combinations are explored, such as syntactic and functional criteria (Cuenca and Crible, 2019), multifunctionality for certain DM clusters (Crible and Degand, 2021; Shirtz, 2021; Koops and Lohmann, 2022). However, the co-occurrence of the three types of DMs is still overlooked. As a result, the current study intends to embark on this perspective and investigate the possibility of combing EDM, CDM, and IDM, but the investigation of reasons is beyond the scope of this study and will not be discussed further here. Apart from this, many comparative studies rely extensively on existing corpora (Müller, 2005; Lam, 2007; Liu, 2017; Hancil, 2018; Huang, 2019; Buysse, 2020; Algouzi, 2021; Koops and Lohmann, 2022) and rarely build their own. This study, guided by the two research questions below, will build three corpora based on TV interviews from China, the US, and the UK to conduct a quantitative analysis of the frequency, patterns, and positions of the three DMs. The examination and comparison of the use of the three DMs in the three corpora will shed light on DMs in greater detail.

1: What are the frequencies, patterns, and positional distributions of the three DMs in the three corpora?
2: What are the similarities and differences (if any) of the three DMs across the three corpora in terms of the above-mentioned aspects?

## Materials and methods

### Corpora of the study

The study uses three corpora of TV interviews from China, the US, and the UK. One representative program is chosen from each of the three countries. The three programs are highly representative with a combination of global vision and unique local characteristics, in which celebrities from various fields are interviewed. Each of the three programs begins with a concise introduction with some background material, and then they move on to the conversation with challenging questions and discussions; the total running time of each episode is no more than 30 mins. *The Point with Liu Xin* has been selected as the Chinese TV interview. The data of this interview consist of two episodes, which are together referred to as the Chinese Corpus. *Amanpour and Company* is a global-news interview program on public broadcasting service (PBS). Two episodes are selected as the sample data, termed the US Corpus. *HARDtalk* is a BBC television and radio program that airs on BBC News Channel. Likewise, the data from this interview also comprise two episodes and is coded as the UK Corpus. The data for this study are randomly extracted from an on-going large-scale project of 120 episodes (almost 3,000 mins). The composition of the three corpora is shown in Table 1 in terms of text code, number of tokens, and proportion of each episode. The data in the present study consist of 20,517 tokens. Due to the balanced sample size and interviewed guests, the three corpora are quite comparable despite their small scale. Each corpus, for example, comprises two interviewees, one of whom is a politician and the other a researcher, resulting in unbiased topics. Furthermore, it is possible to conduct media discourse analysis with small sample size. Cook’s (2014) analysis of politeness and DMs in one episode of a talk show, and Crible and Degand’s (2019) investigation of DM functions in 7,545 words, are two typical illustrations. Therefore, the sample size in this study is acceptable and manageable.

**TABLE 1:** The composition of the three corpora.

	Text code	Number of tokens	Proportion (%)
Chinese Corpus	C_1	3437	52.56
	C_2	3102	47.44
	Total	6539	100.00
US Corpus	A_1	2955	51.12
	A_2	2826	48.44
	Total	5781	100.00
UK Corpus	B_1	4238	49.88
	B_2	4259	50.12
	Total	8497	100.00

### Methodology

**FIGURE 1:**
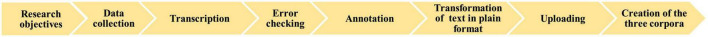
Stages in the development of corpora.

The corpus method complements quantitative and qualitative approaches and often works well and effectively in conjunction with them (Handford, 2015). The application of the corpus technique in the field of pragmatics is both productive and potent due to the automatic search functions (Bonelli, 2010). The availability of corpora has been of great help to recent research on DMs, which has benefited considerably from it. A case in point is the investigation of politeness, hedges, boosters, DMs, deixis, and speech acts (Aijmer and Simon-Vandenbergen, 2004). The three corpora are built individually and make use of analytical tools, such as LancsBox (Brezina et al., 2021) and iFLYTEK’s Hearing App. The first one is for corpus construction, while the second one is for data transcription. LancsBox is a user-friendly, new-generation software package with multiple functions for analyzing corpus and language data. The iFLYTEK’s Hearing App enables multi-terminal, multi-language, multi-scenario, and multi-form voice-to-text transcription. As stated in the last section, six episodes are collected for corpus building, two for each corpus, as the first step. The transcription system, in line with Müller’s (2005), is implemented thoroughly to ensure consistency. After the transcription work is complete, the text needs to be cleaned up because the manually entered text may have some non-standard symbols and formats (Liang et al., 2019). Due to the computer’s inability to detect errors, manual checking is required for verifying each transcription, including spelling, enclitic form, punctuation, anonyms, and proper nouns (Leech, 2005). The following step is to add markup and annotations. Although LancsBox can perform the majority of automatic annotations, some cannot be performed accurately due to the complexity and ambiguity of language (Leech, 2005). For example, syntactic annotation (segmentation) and prosodic annotation (pauses) are conducted. To clearly define the category and identify the corpus, descriptive metadata is presented in a separate file, including the file name, setting, speakers, and length (Reppen, 2010). The names of both the host and the guest are documented so that they may be identified easily. Then the following step is to save the content in a format known as plain text (Wynne, 2005). In the end, each set of texts is uploaded to LancsBox on its own, resulting in a total of three corpora: the Chinese Corpus (6,539 tokens), the US Corpus (5,781 tokens), and the UK Corpus (8,497 tokens). The detailed procedures are outlined in Figure 1. As shown in the subsequent section, a quantitative method is used to compare the three DMs across the three corpora in terms of their frequencies, patterns, and positions. The study uses normalization (Brezina, 2018) and the UCREL log-likelihood test (Rayson, 2016) for the quantitative analysis of the corpora.

### Procedures

The corpora were built for the examination of the frequency, patterns, and position of the three selected DMs, due to the importance of the spoken corpus in DM research (Aijmer, 2015). First, using the KWIC tool in LancsBox (Brezina et al., 2021), a list of all instances of *so, and, but* in the form of concordance lines in the three corpora can be drawn. The corresponding generated concordance lines are saved for subsequent analysis. The frequency of detected DMs is calculated through statistical procedures such as the calculation of the absolute frequency of the three DMs, which include both DMs and non-DM uses, and then the DM frequency is calculated in line with Fraser’s (2009) DM definition and criteria. During this process, cases are excluded if *so* is a pro-form (I guess *so*), degree adverb (*so* good), or in fixed patterns (*so*…*that*); if *and* connects elements below the clause level; if *but* in fixed expressions (*all but*).

Second, all the concordance lines registering with the co-occurrence of the three DMs are extracted *via* the filter function. The co-occurrences can also be visualized using the GraphColl tool, through which collocations are identified and displayed in a collocation graph, which visualizes the collocates’ strength, frequency, and position. The combination of the three DMs can be generated by excluding the non-DM clusters. Using the filter and GraphColl function, the collocation frequency is identified, enabling the investigation of the co-occurrence of the three DMs.

Third, the positional distribution of the three DMs can be observed and counted using the search results for DM frequency. The operational definition of DM position is in line with Fraser’s (2009) and Koops and Lohmann’s (2022) criteria: a complete utterance is a linguistic unit expressing a complete proposition. For example, Fraser (2009) shows that a DM can appear in the initial position (*But*, we arrived on time.), medial position (We, *however*, arrived on time.), and final position (We arrived on time, *however*). Using the KWIC function, the positional distribution of the three DMs in particular concordances in the three corpora was extracted, and their frequency was calculated.

Finally, regarding the comparison of the frequency of the three DMs across the three corpora, normalization is used to allocate the frequency of the specific word to a common basis (Liang et al., 2019). Counts correspond to linear distributions, and normalization is an appropriate methodological choice (Baker, 2006). To enable comparison across corpora of different sizes, the normalized frequency is calculated and 1,000 was used as the common basis for normalization (Brezina, 2018).

The frequency ranking can be calculated by comparing the normalized frequency. The log-likelihood test was used to determine whether there is a significant difference in the frequency of the DM *so, and, but* across the three corpora. The log-likelihood statistic is preferred in frequency comparisons between corpora, as demonstrated in Aijmer’s (2011) study. Using the UCREL log-likelihood wizard (Rayson, 2016), the significance test in frequency between two corpora was performed. Based on the results, the statistical significance can be calculated.

## Results

### Frequency of *so, and, but* in the three corpora

There are 60 instances of *so* in the Chinese Corpus. Forty-five instances are used as DMs, while the other 15 instances are non-DM. The DM *and* occurs 162 times in the corpus, of which 82 instances are excluded; hence, *and* as DM occurs 80 times. There are 31 instances of *but* in total, of which four are excluded; thus, *but* as DM occurs 27 times. The frequency of the three DMs in the Chinese Corpus is shown in Table 2.

**TABLE 2:** The frequency of the DMs *so, and, but* in the three corpora.

	Total	DM	Non-DM	DM (%)
Chinese Corpus				
so	60	45	15	75.00
and	162	80	82	49.38
but	31	27	4	87.10
US Corpus				
so	55	37	18	67.27
and	253	155	98	61.26
but	29	23	6	79.31
UK Corpus				
so	31	22	9	70.97
and	182	87	95	47.80
but	54	47	7	87.04

In the US Corpus, the DM *so* occurs 55 times in total, of which 37 are used as a DM. The DM *and* occurs 253 times in total, with 155 of those instances being used as a DM. There are 29 instances of *but* in the corpus, except for six cases; thus, *but* as DM appears 23 times.

Altogether, Table 2 shows that there are 31 instances of *so* in the UK Corpus, 22 are used as DMs when the remaining nine instances are excluded. There are 182 instances of *and*, of which 87 instances are used as a DM after excluding 95 cases. There are 54 cases of *but*, with 47 cases used as DM when the other seven non-DM uses are excluded.

### Patterns of *so, and, but* in the three corpora

**FIGURE 2:**
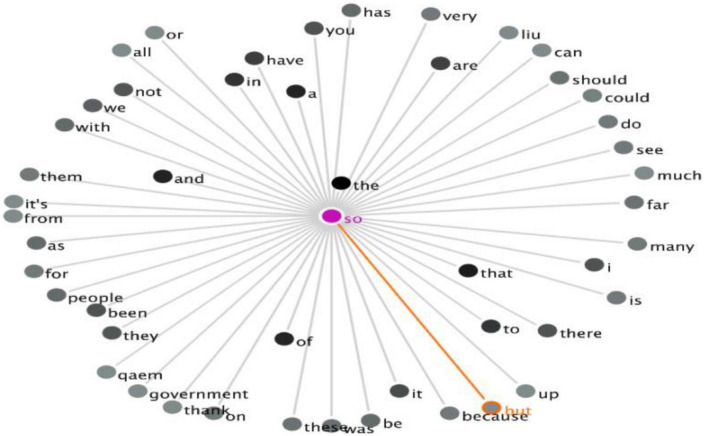
The GraphColl of the co-occurrence of “so but” in the Chinese Corpus.

The patterns discussed in the present study are the co-occurrence/combination of DMs or DM clusters. The frequency of the combination of the three selected DMs in the Chinese Corpus can be seen in Table 3, which demonstrates that there are two co-occurrences (*“so but,” “and so”*) that each occurs only once. In other words, the DM cluster *“and so”* is an example of EDM-IDM, while *“so but”* is a DM cluster of IDM-CDM. The visualization of the combination of *“so but”* is shown in Figure 2.

**TABLE 3:** Co-occurrence of the DMs *so, and, but* in the three corpora.

	DM clusters	Node	Frequency
Chinese Corpus	so but	so/but	1
	and so	so/and	1
US Corpus	and so	so/and	12
	but so	so/but	1
	and but	but/and	1
UK Corpus	and but	and/but	1

**FIGURE 3:**
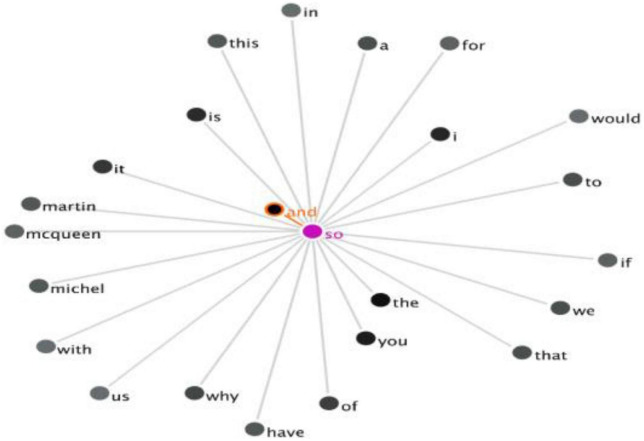
The GraphColl of the co-occurrence of “and so” in the US Corpus.

Similarly, the collocation in the US Corpus can be obtained by employing the filter and GraphColl function. By analyzing the concordance lines with the three DMs as the nodes, 12 instances of EDM-IDM co-occurrence (*“and so”*), one example of CDM-IDM co-occurrence of (*“but so”*), and one instance of the combination of EDM-CDM (*“and but”*) are identified. The number of DM clusters and the collocation of the searched node are shown in Table 3 and are visualized in Figure 3.

**FIGURE 4:**
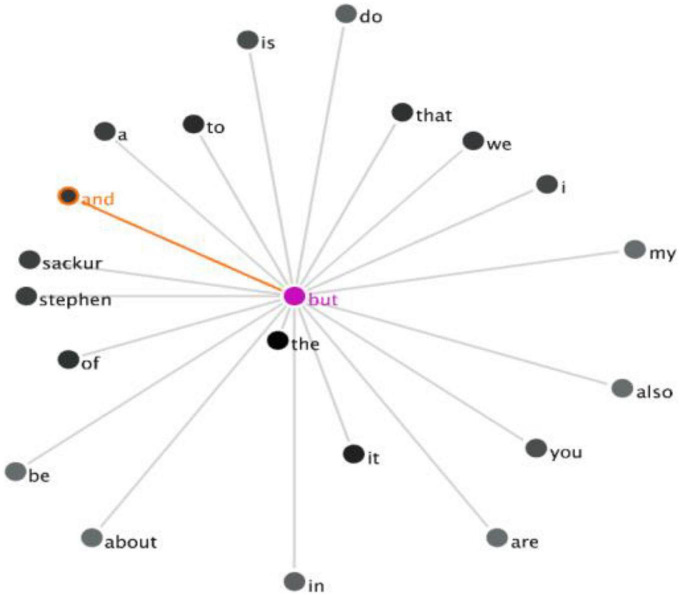
The GraphColl of the co-occurrence of “and but” in the UK Corpus.

The co-occurrence of the DMs *so, and, but* in the UK Corpus can also be obtained by using a similar method. However, no instances of DM clusters were generated when the DM *so* was searched as the keyword. There is only one co-occurrence of the DM cluster *“and but”* in the extracted concordance line. This collocation can be visualized through the GraphColl function in Figure 4.

### Positions of *so, and, but* in the three corpora

Table 4 displays the frequency of the positional distribution of the DMs *so, and, but*. We can see that the three DMs have a similar distribution in terms of the overall position. According to Fraser’s (2009) study, the frequency of appearing in the initial position accounts for a larger proportion, followed by the medial position and the final position. The distribution of the three DMs in the UK Corpus serves as a good illustration.

**TABLE 4:** The frequency of DM position in the three corpora.

	DM	Initial (%)	Medial (%)	Final (%)
Chinese Corpus	so	36 (80%)	9 (20%)	0
	and	39 (48.75%)	41 (51.25%)	0
	but	14 (51.85%)	13 (48.15%)	0
US Corpus	so	29 (78.38%)	8 (21.62%)	0
	and	39 (25.16%)	116 (74.84%)	0
	but	8 (34.78%)	15 (65.22%)	0
UK Corpus	so	20 (90.91%)	2 (9.09%)	0
	and	46 (52.87%)	37 (42.53%)	4 (4.60%)
	but	26 (55.32%)	21 (44.68%)	0

### Comparison of *so, and, but* in the three corpora

A comparison of the frequency of the three DMs in each corpus reveals two common features. One is that the DM *and* occurs more frequently than the other two DMs (*so, but*). The other one is that the frequency ranking of the three DMs is the same in the Chinese Corpus and the US Corpus. For instance, *and* appears 80 times, followed by *so* (45 times) and *but* (27 times) in the Chinese Corpus (Table 2). Similarly, there are 155 instances of the DM *and*, followed by the DM *so* with 37 instances and the DM *but* with 23 instances in the US Corpus (Table 2). A minor distinction in the UK Corpus is the ranking order of the DM *but* and the DM *so*: *but* has a higher frequency (47 times) than *so* (22 times; Table 2). The frequency ranking can be calculated by comparing the normalized frequency (Table 5).

**TABLE 5:** The normalized frequency of the three DMs in the three corpora.

Corpus	Absolute frequency of DM *so*	Number of tokens in corpus	Normalized frequency
Chinese Corpus	45	6,539	6.88
US Corpus	37	5,781	6.40
UK Corpus	22	8,497	2.59

**Corpus**	**Absolute frequency of DM *and***	**Number of tokens in corpus**	**Normalized frequency**

Chinese Corpus	80	6,539	12.23
US Corpus	155	5,781	26.81
UK Corpus	87	8,497	10.24

**Corpus**	**Absolute frequency of DM *but***	**Number of tokens in the corpus**	**Normalized frequency**

Chinese Corpus	27	6,539	4.13
US Corpus	23	5,781	3.98
UK Corpus	47	8,497	5.53

Table 5 above indicates that the DM *so* occurs the most frequently in the Chinese Corpus, followed by the US Corpus and the UK Corpus; the DM *and* has the highest frequency in the US Corpus, followed by the Chinese Corpus and the UK Corpus; and the DM *but* ranks the first in the UK Corpus, followed by the Chinese Corpus and the US Corpus.

The log-likelihood formula shows that the LL (log-likelihood) must be above 3.84 for the difference to be significant at the *p* < 0.05 level. The greater the LL score, the more statistically significant the result. Table 6 shows that there is a statistically significant difference between the Chinese Corpus and the UK Corpus (LL = 15.23) and between the US Corpus and the UK Corpus 3 (LL = 11.81) regarding the frequency of the DM *so*. Regarding the frequency of the DM *and*, there is a statistically significant difference between the Chinese Corpus and the US Corpus (LL = 34.49) and between the US Corpus and the UK Corpus (LL = 54.48). Table 6 shows the results of the significance test of the DM *and* between corpora. However, no statistically significant difference was observed in the frequency of the DM *but* across the three corpora.

**TABLE 6:** The frequency comparison of the DM *so, and* between the corpora.

Corpus	Frequency of the DM *so*	LL	Significance
Chinese Corpus	45	15.23	*p* < 0.05
UK Corpus	22		
US Corpus	37	11.81	*p* < 0.05
UK Corpus	22		

**Corpus**	**Frequency of the DM *and***	**LL**	**Significance**

Chinese Corpus	80	34.49	*p* < 0.05
US Corpus	155		
US Corpus	155	54.48	*p* < 0.05
UK Corpus	87		

The above pairwise comparison displays that there is a statistically significant difference between corpora regarding the use of DM *so*, DM *and*. However, no statistically significant difference is found between corpora in terms of the frequency of DM *but*.

**FIGURE 5:**
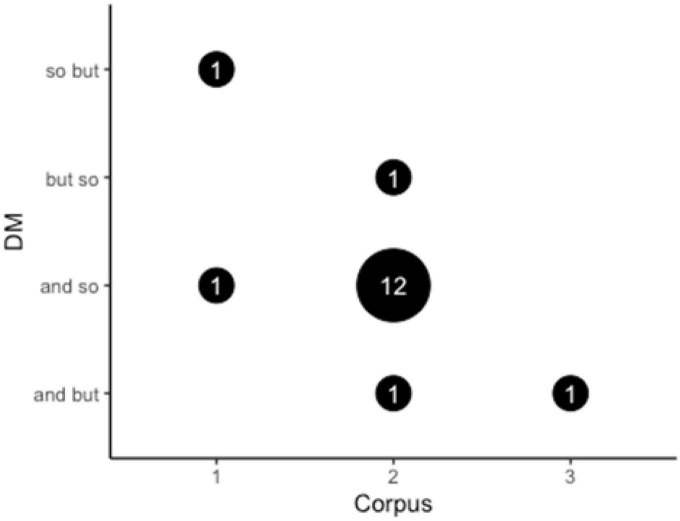
The frequency of the co-occurrences of the three DMs in the three corpora. (1-the Chinese Corpus, 2-the US Corpus, 3-the UK Corpus).

As shown in Table 3, the most frequently occurring combination among the emerging patterns is *“and so,”* which occurs once in the Chinese Corpus and 12 times in the US Corpus. However, it never appears in the UK Corpus. As for the other identified sequencing patterns, *“so but”* only appears once in the Chinese Corpus, *“but so”* only occurs once in the US Corpus, *“and but”* occurs only once in both the US Corpus and the UK Corpus. The comparison of the frequency of the combinations of the three DMs can be visualized in Figure 5.

**FIGURE 6:**
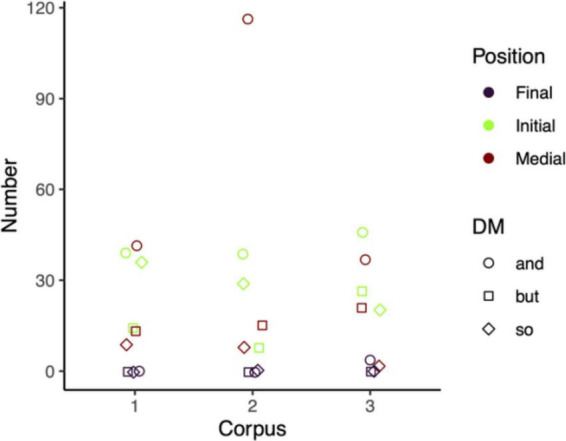
The positional distribution of the three DMs in the three corpora. (1-the Chinese Corpus, 2-the US Corpus, 3-the UK Corpus).

Figure 6 shows that the positional distribution of the DM *so* is quite similar across the three corpora since it has a higher frequency of appearing in the initial position followed by the medial position in all three corpora. As for the DM *and*, the positional distribution in the Chinese Corpus and the US Corpus is quite similar because the DM *and* has a higher frequency in the medial position followed by the initial position, notably in the US Corpus. However, the number of occurrences of the initial position of the DM *and* in the UK Corpus is, however, higher than that in the medial position. Another intriguing feature in the UK Corpus is that only the DM *and* appears four times in the final position. In the US Corpus, the DM *but* has a higher frequency in the medial position followed by the initial position, as seen in Figure 6. This is in contrast to the positional distribution in the other two corpora, where the DM *but* occurs more in the initial position followed by the medial position.

## Discussion

The present study compares the frequency of the three selected DMs, their co-occurrences, and their positions in the three corpora by adopting a corpus-based approach and a quantitative method. The results, to some extent, offer some evidence for the comparability of the three corpora.

First, three DMs are the top three in each of the three corpora in terms of frequency, confirming previous research that they are frequently used in the spoken genre (Carter and McCarthy, 2006). The resemblance can be found between the present study and previous studies (Redeker, 1990; Asik and Cephe, 2013; Crible, 2018) and BNC64 and BNC2014 is that the DM *and* ranks first. However, there are slight differences in the ranking order. For instance, the ranking order of the Chinese Corpus (Table 2) and the US Corpus (Table 2) corresponds to Asik and Cephe’s and Redeker’s studies, as well as spoken BNC64—that is, the DM *and* ranks first, followed by the DM *so* and the DM *but*. Whereas the ranking order of the UK Corpus (Table 2) is consistent with Crible’s (2018) study and BNC64—which shows that the DM *and* ranks first, followed by the DM *but* and the DM *so*. When the frequency of the three DMs is compared across the three corpora, the DM *so* appears the most frequently in the Chinese Corpus (Table 5), the DM *and* occurs the most frequently in the US Corpus (Table 5), and the DM *but* has the highest frequency in the UK Corpus (Table 5). This indicates that inferential expressions associated with the DM *so* are most frequently used in the Chinese interview, elaborative expressions embedded with the DM *and* occur relatively more frequently in the American interview, and contrastive expressions with the DM *but* are used the most in the British interview. In line with previous studies (Lam, 2007; Liu, 2017), some DMs’ observed differences are statistically significant. For example, there is a statistically significant difference between the Chinese Corpus and the UK Corpus (Table 6) and between the US Corpus and the UK Corpus (Table 6) regarding the frequency of the DM *so*. The difference in the rate of the DM *and* between the Chinese Corpus and the US Corpus (Table 6) and between the US Corpus and the UK Corpus (Table 6) also achieves a high statistical significance. However, no statistically significant difference is observed in the frequency of DM *but*. Gender is one of the variables accounting for the frequency of DM use. For example, it is typically women’s language (Östman, 1981). However, the current study cannot draw such firm conclusions because the presented DM frequency is used by both men and women. The Chinese Corpus, for example, has one female host and two male guests; the US Corpus includes one female host, one male guest, and one female guest; and the UK Corpus consists of one male host and two male guests. As a result, further research is required to interpret this phenomenon.

Previous studies have reported that the DM cluster is a frequent phenomenon that is not random and has some discourse-functional motivation (Crible, 2018). Crible and Degand (2021) disentangle some linguistic features that constrain DM clusters and propose a reasonable rule that governs this integration. The three DMs in the present study can be combined to form six co-occurring strings: *“and so,” “and but,” “so but,” “so and,” “but and,” “but so.”* The results (Figure 5) show that four out of the six patterns occur among the three corpora. The combination *“and so”* occurs the most frequently in the US Corpus. Crible’s (2018) study on the frequency of English DM clusters echoes the same finding, revealing the possibility of the combination of EDM and IDM. In contrast to the current study, Lam’s (2007) research does not appear to demonstrate a clear preference for this combination in terms of DM collocations, which is contradictory. Another intriguing aspect is that Fraser’s (2015) study does not find evidence of the possibility of IDM and CDM working together (referred to here as *“so but”*), which is demonstrated in the present study. The results of the patterns reveal a solid tendency for the combination of different types of DMs. Koops and Lohmann’s (2022) general order principle can be used to explain this integration—different DM patterns achieve different effects. In other words, the earlier DM constrains the interpretation of the later one. The DM co-occurrence *“and so”* indicates that the upcoming utterance is a result or conclusion, while *“so and*” marks a topic shift. The one that should be placed first or second is determined by DM functions. The DM sequencing *“and so”* indicates that it is frequently used to start a new topic or turn, particularly in the US corpus. In addition, *“and so”* is regular use in American English (Koops and Lohmann, 2022), which explains the higher frequency of its appearance in American interview. These may be preliminary explanations for speakers’ preference in choosing the DM pattern. Overall, given the frequency of DM clusters in the present study, it does not show a high consistency with previous studies that DM co-occurrence is a frequent phenomenon, or at least it is not as frequent as claimed in previous studies, which may be attributed to the small data set.

Finally, the general positional distribution of the three DMs across the three corpora is quite similar—with the initial position having the highest proportion, followed by the medial position and the final position (Table 4). This is in line with Crible’s (2018) finding that utterance initial is the most typical use of DM followed by utterance medial and final. Moreover, a higher rate of their occurrence in discourse initial position also echoes previous studies (Fraser, 2009; Alsager et al., 2020). Despite the general consistency in the distribution pattern, there is a certain degree of disparity in the proportions of the DM *and* in the Chinese Corpus (48.75 vs. 51.25%) and the US Corpus (25.16 vs. 74.84%) and the DM *but* in the US Corpus (34.78 vs. 65.25%), in which the medial proportion is greater than that of the initial position. As shown in Figure 6, the positional distribution of the DM *so* is less flexible than that of the other two DMs, whose initial position is always the first. This finding, however, contradicts Lam’s (2007) study, which shows that the DM *so* in the utterance medial position constitutes a greater proportion. Moreover, they rarely occur in the utterance final position. Only the DM *and* appears four times (4.6%) in the final position in the UK Corpus (Figure 6). Thus, we may draw a preliminary conclusion that the DM positional distribution does not exhibit a high degree of positional freedom as reported in previous studies (Tanghe, 2016; Bordería and Fischer, 2021). The DM positional distribution can be attributed to different variables. Register plays a crucial role, specifically in the degree of interactivity: the more interactive the register, the more frequently it occurs in the initial position (Crible, 2018). Three interviews are all interactive, interpreting their higher proportion of the occurrence in the initial position. In addition, their rare appearance in the final position is also due to the limited number of tag questions in the three corpora (Crible, 2018). More thorough larger-scale research is required to better understand this phenomenon.

## Conclusion

The present study examined and compared the frequency, patterns, and positional distribution of the three DMs in the three corpora. The study concluded that there are statistically significant differences and marginal variations within and between corpora in different aspects. The study differed most significantly from previous ones in terms of the data type and objectives. While previous studies concentrated more on qualitative analysis of DM functions in academic settings or only one of the above-mentioned aspects, this study attempted to investigate all three aspects in one single goal in media discourse from a quantitative perspective. The study revealed that DM *so* occurs most frequently in the Chinese Corpus; the US Corpus has the highest frequency of the DM *and*; and the UK Corpus has the highest frequency of DM *but*, which provides a basis for a more productive analysis of the types of DMs used in media talk. Do all IDMs, for example, appear frequently in Chinese media discourse? Or are there more EDMs in American interviews? Or are CDMs more likely to occur in British media discourse? The article found that the DM cluster *“and so”* is most frequently used in American interviews, confirming that this combination is typical use in American English. The higher frequency of DMs appearing in the initial position also confirmed that the more interactive the genre, the more likely DMs appear in the initial position.

Hence, the study contributes to the existing literature by filling the gap and adding more insights into the field of discourse markers. Apart from academe, the study also sheds light on the role of DMs in interviews, which may inspire hosts and guests to consider how to achieve the best bilateral communication by using certain DMs. Despite the foregoing insights, this study falls short of expectations and does have some limitations. The findings in the present study cannot be generalized due to the small data set. Although this study provides some preliminary explanations for the differences, it fails to account for more specific variables due to the small sample size and the scope of this study. This opens new avenues for the future research in encompassing both the large corpus and another genre in order to address this problem. It is intended that the results presented in this study would be of benefit not only to individuals who conduct interviews for the media and those who are interviewed for the media, but also to those who aspire to be successful doing DM research in media settings.

## Data availability statement

The raw data supporting the conclusions of this article will be made available by the authors, without undue reservation.

## Author contributions

Both authors listed have made a substantial, direct, and intellectual contribution to the work, and approved it for publication.
